# Xenosurveillance: A Novel Mosquito-Based Approach for Examining the Human-Pathogen Landscape

**DOI:** 10.1371/journal.pntd.0003628

**Published:** 2015-03-16

**Authors:** Nathan D. Grubaugh, Supriya Sharma, Benjamin J. Krajacich, Lawrence S. Fakoli III, Fatorma K. Bolay, Joe W. Diclaro II, W. Evan Johnson, Gregory D. Ebel, Brian D. Foy, Doug E. Brackney

**Affiliations:** 1 Department of Microbiology, Immunology, and Pathology, Colorado State University, Fort Collins, Colorado, United States of America; 2 Division of Computational Biomedicine, Boston University School of Medicine, Boston, Massachusetts, United States of America; 3 Liberian Institute for Biomedical Research, Charlesville, Liberia; 4 United States Naval Medical Research Unit No. 3, Cairo, Egypt; Aix Marseille University, Institute of Research for Development, and EHESP School of Public Health, FRANCE

## Abstract

**Background:**

Globally, regions at the highest risk for emerging infectious diseases are often the ones with the fewest resources. As a result, implementing sustainable infectious disease surveillance systems in these regions is challenging. The cost of these programs and difficulties associated with collecting, storing and transporting relevant samples have hindered them in the regions where they are most needed. Therefore, we tested the sensitivity and feasibility of a novel surveillance technique called xenosurveillance. This approach utilizes the host feeding preferences and behaviors of *Anopheles gambiae*, which are highly anthropophilic and rest indoors after feeding, to sample viruses in human beings. We hypothesized that mosquito bloodmeals could be used to detect vertebrate viral pathogens within realistic field collection timeframes and clinically relevant concentrations.

**Methodology/Principal Findings:**

To validate this approach, we examined variables influencing virus detection such as the duration between mosquito blood feeding and mosquito processing, the pathogen nucleic acid stability in the mosquito gut and the pathogen load present in the host’s blood at the time of bloodmeal ingestion using our laboratory model. Our findings revealed that viral nucleic acids, at clinically relevant concentrations, could be detected from engorged mosquitoes for up to 24 hours post feeding by qRT-PCR. Subsequently, we tested this approach in the field by examining blood from engorged mosquitoes from two field sites in Liberia. Using next-generation sequencing and PCR we were able to detect the genetic signatures of multiple viral pathogens including Epstein-Barr virus and canine distemper virus.

**Conclusions/Significance:**

Together, these data demonstrate the feasibility of xenosurveillance and in doing so validated a simple and non-invasive surveillance tool that could be used to complement current biosurveillance efforts.

## Author Summary

The emergence of infectious diseases can have devastating public health and economic consequences. Being able to identify such events at early stages could significantly mitigate some of these outcomes. In many resource poor regions of the world, current surveillance methods are hindered by the costs associated with collecting, storing and shipping clinical specimens. Herein we present a novel surveillance approach termed xenosurveillance that addresses some of these hurdles. Specifically, we leverage the feeding behavior of mosquitoes to sample human blood, which is then preserved on filter cards. Our laboratory studies demonstrated that such an approach can be used to detect vertebrate viral pathogens within realistic field collection timeframes at clinically relevant concentrations. Subsequently, we tested the feasibility of xenosurveillance in West Africa and found the presence of two vertebrate viral pathogens, Epstein Barr virus and canine distemper virus. Together, these data demonstrate the feasibility of xenosurveillance and suggest that it could be used to detect and predict pathogen outbreaks at earlier, more actionable stages of emergence.

## Introduction

Over the last half century infectious diseases have emerged with increasing frequency [1]. Emerging infectious diseases (EIDs) have profound public health and economic consequences as highlighted by the ongoing epidemics of Ebola virus in West Africa [2], MERS coronavirus in the Middle East [3], and chikungunya and West Nile viruses in the Americas [4]. The emergence of these diseases can, in large part, be attributed to the increased frequency and duration of human and livestock interactions with wildlife populations [5]. Greater than 60% of EIDs since 1940 are due to zoonotic infections [1]. Biosurveillance efforts now focus on crucial interfaces (i.e. transmission between animals, initial human spillover events and localized emergence) in order to identify pathogens with epidemic and pandemic potential [6,7]. Yet allocation of resources are not always directed towards predicted emerging disease ‘hotspots’ like tropical Africa where relatively few EID events have been documented locally [1]. This is mainly due to difficulties in sample collection, storage and transport from remote and underdeveloped parts of the world. Although a wide array of assays exist to detect emerging pathogens (e.g. [8–10]), novel and inexpensive methods for sample collection and for pathogen detection are needed to use them most effectively.

The over 3000 described species of mosquitoes are found on every continent except Antarctica. A large majority of these are hematophagous and rely on vertebrate blood for nourishment and reproduction. *Anopheles gambiae* sensu stricto is the major human malaria vector in Africa. This species is highly anthropophilic (>95% of bloodmeals come from humans when control measures are absent), feeds frequently, and prefers to blood feed at night and often in human dwellings [11]. After blood feeding, these mosquitoes rest on interior walls for several hours digesting their newly acquired bloodmeal before they exit the dwelling. While engorged, their mobility is limited, and it is at this vulnerable point that they can be easily collected via aspiration and bloodmeals analyzed for the presence of pathogens, a process we have termed *xenosurveillance*. Recently, Kading et al. demonstrated that mosquitoes could be used as ‘biological syringes’ to accurately quantify viremias in animals [12]. In fact, two recent field studies have demonstrated that vertebrate viral pathogens that are not vector-borne could be detected in the bloodmeals of *Culicidae* mosquitoes [13,14]. These findings suggest that hematophagous insects, specifically mosquitoes, could make sample acquisition and pathogen surveillance more tractable in remote tropical locales.

Several important questions remain to be answered when evaluating the ultimate utility and feasibility of xenosurveillance. Specifically, variables influencing pathogen detection such as the duration between mosquito blood feeding and mosquito processing, the pathogen nucleic acid stability in the mosquito gut and the pathogen load present in the host’s blood at the time of bloodmeal ingestion remain to be determined. Therefore, we examined some of these variables and hypothesized that mosquito bloodmeals could be used to detect animal pathogens within realistic field collection timeframes and clinically relevant concentrations. We tested our hypothesis using *An*. *gambiae* mosquitoes and, because at least a quarter of human emerging pathogens are viruses [1], viruses from four evolutionarily distinct families, none of which are vectored by *An*. *gambiae* mosquitoes. To make this approach more tractable in remote tropical regions, we preserved the bloodmeals onto Flinders Technology Associates (FTA) filter paper cards for subsequent nucleic acid detection [15–17]. Finally, we collected *An*. *gambiae* bloodmeals from houses in two remote villages in northern Liberia and analyzed them by next generation sequencing (NGS) and PCR to validate our methods. It should be noted that the use of highly anthropophilic *An*. *gambiae* mosquitoes may preclude the identification of potentially zoonotic viruses before they cross the species barrier; however, it will enable the detection of pathogens that have already gained the ability to infect humans but that remain undetected due to limited infection and spread, subclinical illness or clinical misdiagnoses. Our results suggest that xenosurveillance can be used in high-risk tropical regions to detect pathogen outbreaks at early stages of emergence.

## Materials and Methods

### Ethics statement

Experiments involving mosquitoes and animals (protocol 12–3687A) were approved by the Colorado State University (CSU) Institutional Animal Care and Use Committee. The protocol adhered to the guidelines outlined in the Animal Welfare Act Regulations (CSU IACUC: OLAW Animal Welfare Assurance Number: A3572–01). Mosquito collections from houses in Liberia were reviewed and approved by the Liberian Institute for Biomedical Research (EC/LIBR/012/034) and CSU (11–3121H), and they were performed with the consent of each head of household and the community.

### Viruses and mosquitoes

Four viruses that are not vectored by *An*. *gambiae* mosquitoes (they do not infect these mosquitoes, nor can they be transmitted by these mosquitoes) were used for these studies: human immunodeficiency virus 1 (HIV-1, *Retroviridae*) strain NL4–3 passaged in SUP-T (human T lymphoblast) cells, West Nile virus (WNV, *Flaviviridae*) strain NY99 derived from an infectious clone as previously described [18], pirital virus (PIRV, *Arenaviridae*), BSL-3 rodent model for Lassa virus, strain VAV-488 passaged in Vero E6 (African green monkey kidney epithelial) cells and chikungunya virus (CHIKV, *Togaviridae*) strain LR2006-OPY1 passaged in Vero E6 cells. *Anopheles gambiae sensu stricto* G3 mosquitoes were maintained at 28 ± 2°C, 80% humidity and under a 14:10 (L:D) photoperiod. Adult mosquitoes were provided with water and 10% sucrose solution *ad libitum* and were used in experiments 4–7 days post-emergence.

### Mosquito blood-feeds

**Fig 1:**
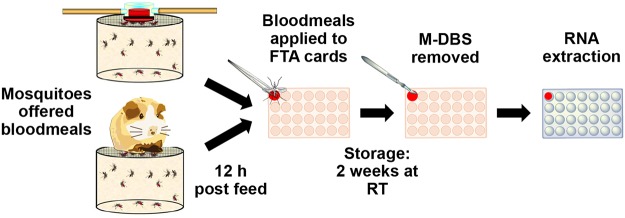
Laboratory preparation of mosquito bloodmeals for detection of viral RNA. Mosquitoes were offered bloodmeals from artificial membrane feeders containing virus-spiked sheep’s blood or virus-infected hamsters. The bloodmeals were applied to FTA cards 12 h post blood feeding, unless otherwise indicated. The FTA cards were left to air dry for 2 hours and stored at room temperature for 2 weeks. The mosquito dried blood spots (M-DBS) were removed from the FTA cards and RNA was extracted.

An overview of the experimental design is shown in Fig. 1. Briefly, known concentrations of virus mixed with blood were used to determine if viral RNA could be detected in mosquito bloodmeals. The viruses were diluted in cell-culture medium (Eagle’s minimum essential medium containing 10% heat-inactivated fetal bovine serum) to twice the desired RNA copies/ ml concentration and mixed equally with defibrinated sheep’s blood (Colorado Serum Company, Denver, CO) to reach the final desired concentration. Water and sucrose were removed from mosquitoes 12 hours prior to blood feeding. Mosquitoes were offered virus-containing blood via an artificial feeding apparatus. Back titrations of the bloodmeals were prepared by directly applying 2 μl of the virus-blood mix (the approximate volume of an *Anopheles* bloodmeal) directly onto CloneSaver FTA cards (GE Healthcare Life Sciences, Little Chalfont, United Kingdom) and diluted with 48 μl of sterile phosphate buffered saline (PBS) for RNA extraction.

### Hamster infection and live animal mosquito blood-feeds

Syrian golden hamsters (*Mesocricetus auratus*) have been previously described as animal models for both PIRV and WNV infection [19,20]. These models were used to determine if viral RNA could be detected from mosquito bloodmeals acquired from active viral infections. Six female hamsters (Charles River Laboratories, Wilmington, MA), weighing 169–184 g at 11 weeks old were inoculated intraperitoneally with 0.1 ml of 5 x 10^6^ RNA copies of PIRV or 5 x 10^4^ RNA copies of WNV (three hamsters for each virus). On days two, four and six post infection, the hamsters were anesthetized with an intramuscular injection of 100 mg/ kg of ketamine and 5 mg/ kg xylazine and 10–50 μl of blood was collected via the jugular vein. Aliquots of hamster blood (2 μl) were applied to FTA cards to determine circulating virus titers. While the hamsters were still anesthetized, they were placed on cartons containing 20–30 mosquitoes and the mosquitoes were allowed to blood feed on them for up to 30 minutes.

### Bloodmeal collection and storage on FTA cards

At the desired time post blood feeding, the mosquitoes were anesthetized by triethylamine and the blood from engorged mosquitoes were collected on FTA cards as follows: 1) forceps clutching the thorax held the mosquito into position on the FTA card, 2) a pipet with a sterile tip pressed the bloodmeal out of the abdomen, 3) the bloodmeal was lightly spread onto the FTA card to make a 3 to 4 mm diameter circle and 4) 5 μl of sterile PBS was applied to the bloodmeal to facilitate diffusion into the FTA card. The FTA cards were dried at room temperature for 3 h, placed into CloneSaver multi-barrier pouches (GE Healthcare Life Sciences) containing desiccant, and stored at room temperature for 2 weeks to model approximate storage time of field collected bloodmeals.

### Viral RNA extraction

Extraction of viral RNA from dried blood spots (DBS) stored on FTA cards were based on previously described methods [17]. A Harris 3 mm micro-puncher (GE Healthcare Life Sciences) was used to remove 1 to 3 punches of the DBS on FTA cards and the punches were placed into a 96-well plate with 70 μl of RNA rapid extraction solution (Ambion, Austin, TX). The puncher was cleaned with 70% ethanol between samples. The 96-well plate was covered with an adhesive plate sealer and placed on a plate rocker for 20 min. at room temperature. Viral RNA was isolated from 50 μl of RNA rapid extraction solution containing FTA card punches or directly from blood diluted in PBS (back titration samples) using the MagMAX-96 viral RNA isolation kit (Ambion) and the KingFisher Flex Magnetic Particle Processor (Thermo Fisher Scientific, Waltham, MA) according to vendor’s protocols. The RNA was eluted in 50 μl of nuclease-free water.

### Quantification of viral RNA copies

TaqMan-based RNA copy standards were transcribed from WNV envelope gene (1031–3430 nt) [21], HIV-1 gag gene (527–1423 nt), PIRV nucleocapsid gene (1762–2400 nt) [22] and CHIKV nonstructural 2B gene (2701–3435 nt) PCR amplicons using the T7 Megascript kit (Ambion). In-house designed and previously described [23] primers and probes were used for qRT-PCR (S1 Table). The qRT-PCR assays were performed in 25 μl reactions using the iScript One-step RT-PCR Kit for probes (Bio-Rad Laboratories Inc., Hercules, CA) and qRT-PCR amplification was carried out using a standardize program at 50°C for 20 min, 95°C for 5min, and 40 cycles of 95°C for 10 s and 60°C for 1 min. Fluorescence was read at the end of the 60°C annealing-extension step. The CFX96 Real-Time instrument using CFX manager software was used for qRT-PCR amplification, data acquisition, and analysis (Bio-Rad Laboratories Inc.). The assay lower limits of quantification (LLOQ) for 2 μl of blood is 20 RNA copies, or 10^4^ RNA copies/ml.

### Field collection of indoor resting blood fed mosquitoes

The field study was conducted in June of 2013 in two rural villages in northern Liberia (near boarders of Sierra Leone and Guinea). Village A had 31 occupied houses with a population of <150 people (all houses used in this study). Village B had a population >3000 people and 12 houses were used in this study. Battery powered Insecta-Zooka (BioQuip, Rancho Dominguez, CA) aspirators were used to collect mosquitoes resting on the walls, ceilings and bed nets inside of the village houses (S1A Fig.). Using mouth aspirators (Model 412; John W. Hock Company, Gainesville, FL), mosquitoes were transferred from the collection cups to 16 oz cardboard containers screened with organdy (and labeled with date and location) and immediately transported to a field lab (S1B Fig.). For long-term anesthesia, the mosquito cartons were placed inside of a plastic bag container containing a wick dipped in triethylamine for 1–2 min (S1C Fig.). The mosquitoes were sorted by blood feeding status (S1D Fig.), and all aspirated mosquitoes were identified as *An*. *gambiae* sensu stricto as previously described [24]. Forceps clutching the thorax were used to hold blood fed mosquitoes in place while the contents of the abdomen were pressed onto 96-well format FTA cards (one blood meal per “well”) using sterile pipet tips (S1E Fig.). The forceps were cleaned with 70% ethanol between mosquitoes. The blood meals were gently spread using the pipet tip and 2–5 μl of ethanol was added to help diffuse the blood into the card (5 μl of sterile PBS is an alternative option, see S2 Fig.). The FTA cards were sealed immediately in multi-barrier pouches containing desiccant to dry the blood spots (S1F Fig.). The desiccant was changed every day for three days then the pouches were stored in a cool, dry place for 1–3 weeks until being transported to CSU, whereupon they were stored at -80°C until RNA extraction. In village A, seven collections occurred every other day for two weeks and each house was aspirated every other collection. In village B, four collections occurred every four to six days and each study house was aspirated every collection.

### Deep sequencing of field-collected mosquito bloodmeal-derived DBS

RNA extracted from individual mosquito bloodmeal-derived DBS (M-DBS, 30 μl each) were pooled together (n = 24–57). The pooled RNAs were concentrated to a volume of 20 μl by precipitation with sodium acetate and ethanol followed by DNase treatment (Ambion) and RNA purification using Agencourt RNAclean XP beads (Beckman Coulter Genomics, Pasadena, CA). The RNA pools were prepared for NGS using the NuGEN Ovation RNA-Seq System V2 (San Carlos, CA) and Nextera XT DNA Sample Preparation Kit (Illumina, San Diego, CA) according to the manufacturer’s recommendations. Each library was constructed with a unique barcode and subsequently combined into pools of four and sequenced on two lanes of the Illumina HiSeq (Beckman Coulter Genomics, Danvers, MA).

### Sequencing analysis

Sequencing data was processed using several modules from the PathoScope 2.0 software framework [25,26]. PathoScope provides a complete bioinformatics framework for quantifying the proportions of reads from individual microbial strains present in sequencing data from samples from environmental or clinical sources. The individual PathoScope pipeline modules perform many computational analysis steps; including reference genome library extraction (PathoLib), read quality control (PathoQC), alignment (PathoMap), strain identification (PathoID), and summarization and annotation of the results (PathoReport). While the bioinformatics approach implemented in this study is sufficient for identifying known viruses, additional pipelines may be added to facilitate the discovery of novel viruses.

We first used the PathoLib module to generate reference libraries (S2 Table). These libraries consisted of all reference sequences from the NCBI nucleotide database (http://www.ncbi.nlm.nih.gov/nucleotide/; download date: 09/2013) that matched the provided NCBI taxonomy levels, as well as all reference sequences in lower levels of the NCBI taxonomy tree. To process the sequencing reads, the PathoQC module was first used to conduct quality control and filtering prior to alignment, including the removal of adapter sequences, read duplicates, and low quality bases/reads. The PathoMap module was used to align the reads to the reference libraries (S2 Table) and also to filter the reads that aligned to the mosquito reference library. PathoID was then used to reassign ambiguously aligned reads and PathoReport was used to annotate the results and generate contiguous sequences from the reads that aligned to each genome. The specific parameters used for each of the PathoScope modules are detailed in S3 Table. 100bp reads were aligned to the Epstein-Barr virus (EBV, GenBank V01555.2) and canine distemper virus (CDV, GenBank KM280689.1) genomes using MOSAIK [27].

### Confirmation of sequencing results

EBV and CDV RT-PCR were performed using conventional methods (primers are listed in S1 Table) and the amplicons were visualized on 1% agarose gels stained with ethidium bromide. PCR amplicons were sequenced using the ABI 3100 genetic analyzer (Thermo Fisher Scientific), and the sequenced nucleotides were analyzed using Geneious version 7.0.6. (Biomatters Limited, New Zealand). Bloodmeals were identified by multiplexed PCR targeting cytochrome b DNA as described [28].

### Statistical analyses

Recovery of viral RNA detection using FTA cards and mosquito bloodmeals was analyzed for each virus by a one way ANOVA with Tukey’s multiple comparisons adjustment. The duration of viral RNA detection from bloodmeal ingestion to application to FTA cards was analyzed for each virus by a one way ANOVA with Dunnet’s multiple comparisons adjustment. Viral RNA recovery from artificial and hamster M-DBSs were compared by unpaired t tests. All statistical analyses were performed in GraphPad Prism version 6 for Windows (GraphPad Software, San Diego, CA).

## Results

### Efficiency and duration of viral RNA recovery from M-DBSs

**Fig 2:**
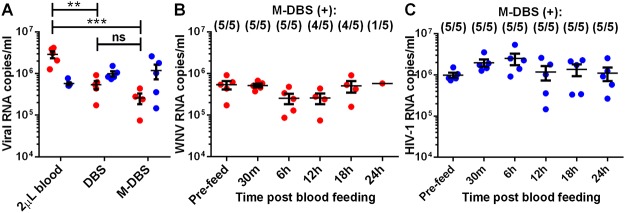
Virus-specific differences between the efficiency and duration of viral RNA detection from M-DBS. WNV (red) or HIV-1 (blue) were mixed with blood at 10^6^ viral RNA copies/ml. (A) The means (shown with SEM) of virus-specific RNA detected directly from 2 μl of blood-virus mix (not applied to FTA cards), 2 μl of the blood-virus mix applied to FTA cards and mosquito bloodmeals applied to FTA cards 12 h post feeding (hpf) were compared using a one way ANOVA with Tukey’s multiple comparisons adjustment. Application of the blood-virus mix (P = 0.0012) and mosquito bloodmeals (P = 0.0008) to FTA cards resulted in significant losses in WNV RNA detection compared to the original blood-virus mix, but not between the treatments applied to FTA cards. Comparisons between HIV-1 treatments were not significant. (B) WNV or (C) HIV-1 mixed with blood were offered to mosquitoes and the bloodmeals were applied to FTA cards at the indicated times post blood feeding. The blood-virus mixes (2 μl each) were applied to FTA cards before blood feeding (pre-feed) to compare to viral RNA detection from mosquito dried blood spots (M-DBS). The number of blood spots positive (>10^4^ RNA copies/ml) for virus RNA per dried blood spot tested (n = 5) are shown above each time point. The means (shown with SEM) of the post blood feeding time points were compared to the pre-feed using a one way ANOVA with Dunnet’s multiple comparisons adjustment. The comparisons were not significant.

Blood offered to mosquitoes, DBSs (i.e. blood dried on FTA cards) and M-DBSs (i.e. bloodmeal expelled from the mosquito and dried on FTA cards) were compared to quantify losses in sensitivity associated with mosquitoes and FTA cards (Fig. 2). Blood containing approximately 10^6^ RNA copies/ml of WNV or HIV-1 were offered to mosquitoes using artificial membrane feeders. At the same time, 2 μl of the virus-spiked blood (approximate volume of a mosquito bloodmeal) were diluted in phosphate buffered saline (PBS) (to determine the pre-blood feed concentration) and applied directly onto FTA cards (to determine the recovery of FTA cards alone). Twelve hours post feeding (hpf) the mosquito bloodmeals were expelled onto FTA cards. WNV and HIV-1 RNA were quantified by qRT-PCR to compare viral RNA recovery (Fig. 2A). The concentration of HIV-1 RNA from DBS and M-DBS remained unchanged compared to the pre-blood feed concentration. There were significant losses in WNV RNA recovery from blood applied onto FTA cards (5.4 fold reduction) and mosquito bloodmeals pressed onto FTA cards (11.2 fold reduction and only 4/5 > LLOQ) compared to the pre-feed blood. However, an insignificant reduction was measured between the DBSs and M-DBSs, indicating that the majority of RNA loss was due to WNV storage on FTA cards. There were not significant differences in viral and total RNA recovery between using PBS or ethanol to apply bloodmeals to FTA cards (S2 Fig).

Mosquitoes provided a virus-spiked bloodmeal were sampled over a 24 h period in order to establish the temporal stability of viral RNA in mosquito guts and determine whether degradation of viral RNA in mosquitoes would likely occur prior to mosquito sampling. Mosquitoes were fed blood mixed with approximately 10^6^ RNA copies/ml of WNV or HIV-1 and the bloodmeals of engorged mosquitoes were collected on FTA cards at 30 min, 6 h, 12 h, 18 h and 24 h post feeding (n = 5 each). Blood spiked with WNV or HIV-1 (2 μl, n = 5 each) were applied to FTA cards before being fed to mosquitoes to determine the pre-blood feed virus concentration. RNA copies were quantified from the DBS and M-DBS for all time points pre- and post-blood feeding (Figs. 2B-2C). The number of M-DBS with detectable amounts of WNV RNA decreased over time. WNV RNA could be detected from all of the M-DBS through 6 hpf, from 4/5 bloodmeals at 12 and 18 hpf and from only 1/5 bloodmeals at 24 hpf (Fig. 2B). However, the quantity of WNV RNA in positive mosquitoes did not significantly decrease over time. HIV-1 RNA was detected in all the M-DBS tested and the RNA copies remained constant over the 24 hour sampling period (Fig. 2C). Mosquito bloodmeals older than 24 hpf were considerably more viscous (and darker in color) making them less effectively absorbed into the FTA cards.

### Viral RNA limits of detection from mosquito bloodmeals

We used ten-fold serial dilutions of four distinct viruses in artificial bloodmeals to determine whether xenosurveillance could effectively detect viral pathogens within clinically relevant ranges. WNV, HIV-1, PIRV or CHIKV spiked blood (ranging from 10^8^ to 10^4^ RNA copies/ml) were fed to mosquitoes and harvested on FTA cards 12 hpf. Using qRT-PCR, we defined the 50% detection limits for each virus from M-DBS using the Reed-Muench method [29]. These data were then compared to published reports of viral loads in patients (Table 1). Because PIRV is not a known human pathogen, we used Lassa fever virus, a human pathogenic arenavirus, for clinical comparisons. Concentration-dependent increases in detection were measured for each virus. None of the viruses were detected in M-DBS from mosquitoes that fed on blood containing 10^4^ viral RNA copies/ml because the approximately 10–30 RNA copies present in 1–3 μl bloodmeals fell below the qRT-PCR assays detection limits.

**Table 1:** Mosquito bloodmeal limits of detection and comparisons to clinical ranges.

	Bloodmeal source RNA copies/ml (n positive/n bloodmeals tested)					50% endpoint detection (RNA copies/ml)	Clinical range (RNA copies/ml)
Virus	10^4^	10^5^	10^6^	10^7^	10^8^		
WNV	0/5	1/5	4/5	5/5	5/5	3.2 × 10^5^	10^3^–10^5^ [30]^a^
HIV-1	0/5	5/5	5/5	5/5	n.t.	3.2 × 10^4^	10^4^–10^7^ [31]
PIRV	0/5	0/5	4/5	5/5	n.t.	4.2 × 10^5^	10^9^ [8,32]^b^
CHIKV	n.t.	0/5	0/5	2/5	3/5	3.2 × 10^7^	10^5^–10^12^ [33,34]

^a^Data from blood donors.

^b^Clinical data for Lassa fever virus. WNV, West Nile virus; HIV-1, human immunodeficiency virus-1; PIRV, pirital virus; CHIKV, chikungunya virus; n.t., not tested.

### Detection of viral RNA from infected hamsters

**Fig 3:**
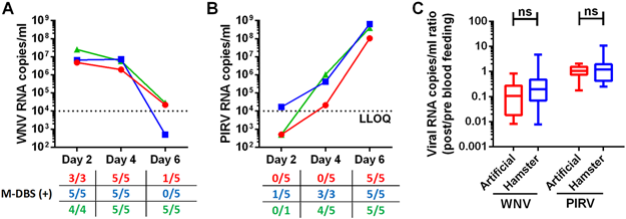
Detection of viral RNA from infected hamsters is not significantly different than from artificial bloodmeals. Hamsters were infected with (A) WNV or (B) PIRV and blood was collected on days 2, 4 and 6 post infection to determine viral RNA copies/ml (n = 3 represented by different colors shown in graphs). Prior to blood collection, the hamsters were offered to mosquitoes as bloodmeals. The tables show the number of mosquito dried blood spots (M-DBS) that were positive for virus-specific RNA, determined by qRT-PCR titers greater than the lower limit of quantification (LLOQ), per M-DBS tested for each hamster and time point. Table text colors represent different hamsters offered as bloodmeals and correspond to the colors in the graphs. (C) Ratios of viral RNA titers (means with min to max whiskers) post blood feeding (M-DBS) to pre blood feeding (2 μl blood applied to FTA cards) were used were used to determine the recovery of viral RNA from mosquito bloodfeeding. The post/pre blood feeding ratios between virus-spiked artificial and infected hamster bloodmeal sources were compared using unpaired t tests and were not significant.

We next assessed whether bloodmeals acquired from active replicating infections in hamsters altered xenosurveillance sensitivity compared to artificial membrane feeds (Fig. 3). Hamsters were infected with either PIRV or WNV (n = 3 each). Mosquitoes were allowed to feed on the hamsters 2, 4 and 6 days post infection (dpi) and held for 12 hpf before applying the bloodmeals to FTA cards. Prior to mosquito feeding, 2 μl of blood were collected and applied to FTA cards to quantify viral load at the time of mosquito exposure. WNV RNA could be detected in all of the M-DBS on days 2 and 4 dpi when circulating hamster WNV titers were > 10^6^ RNA copies/ ml, but as circulating WNV titers approached or fell below the LLOQ (10^4^ viral RNA copies/ ml for 2μl of blood) at 6 dpi, WNV RNA could be detected in only 6/15 M-DBS (Fig. 3A). Hamsters infected with PIRV had low circulating titers during early infection (< 10^4^ RNA copies/ ml on 2 dpi, <10^6^ RNA copies/ml on 4 dpi) and therefore PIRV could only be detected from 1/11 and 7/13 M-DBS from 2 dpi and 4 dpi, respectively (Fig. 3B). On 6 dpi, when the hamster PIRV titers were >10^8^ RNA copies/ml, 15/15 M-DBS were positive for PIRV RNA.

Data from all of the WNV and PIRV experiments were compiled together to further compare the recovery of viral RNA from mosquitoes taking bloodmeals from artificial membranes and infected hamsters. Ratios of viral RNA titers post blood feeding (mosquito bloodmeals applied onto FTA cards) to pre blood feeding (2 μl blood applied onto FTA cards) were used to make comparisons between recovery of RNA from artificial and hamster bloodmeals (Fig. 3C). A ratio <1 indicates a loss of viral RNA recovery through the use of mosquito bloodmeals. From both artificial blood meals and those from infected hamsters, the WNV RNA recovery ratio was <1, indicating that WNV RNA recovery from M-DBS was less efficient than from DBS; although not significantly (Fig. 2A). Similarly, M-DBS acquired from actively replicating virus in hamsters did not significantly differ from WNV-spiked blood in an artificial feeder. In contrast, the recovery ratio of PIRV RNA was approximately 1 and also did not differ between the two blood feeding methods (Fig. 3C).

### Sequencing of bloodmeals from mosquitoes collected in Liberia

**Fig 4:**
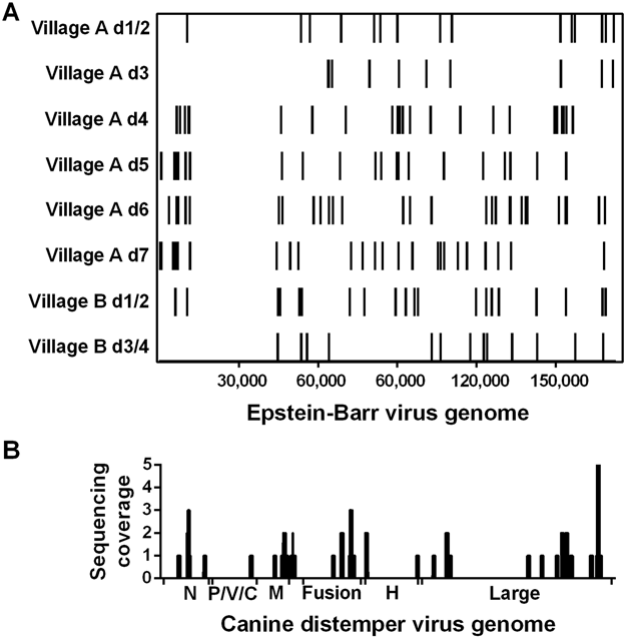
Sequencing reads from mosquito bloodmeals collected in Liberia aligning to Epstein-Barr virus (EBV) and canine distemper virus (CDV) genomes. Bloodmeals from indoor resting mosquitoes were collected from two villages in northern Liberia and sequenced. (A) All pooled M-DBSs contained 100bp reads aligning to EBV and (B) one pool of M-DBSs (Village A d7) contained 100bp reads aligning to CDV. N, nucleoprotein; P/V/C, phospho- and nonstructural V and C proteins; M, matrix protein; H, hemagglutinin protein.

Indoor resting, bloodfed mosquitoes were collected in northern Liberia to assess the feasibility of xenosurveillance for the detection of human pathogens in remote areas with severe resource constraints (Table 2). The PathoScope alignment summaries to the reference genome libraries are shown in Table 3. NGS analysis of the microbial composition in bloodfed *An*. *gambiae* collected within homes in rural Liberia revealed the presence of numerous mosquito midgut microbiota (e.g. *Acinetobacter* and *Pantoea*) and human skin-associated microbes (e.g. *Staphylococcus epidermidis* and *Propionibacterium acnes*) (S3 Fig), some as detected previously [35]. In addition, genetic signatures for EBV were identified in all eight field-derived M-DBS pools, but not the control pool generated from laboratory mosquitoes that had fed upon defibrinated sheep’s blood. Recovered EBV reads were distributed across the length of the genome (Fig. 4) and displayed varying levels of abundance (Table 3). This result was confirmed by using RT-PCR to detect EBV-encoded RNA 2 (highly abundant EBV transcripts during latent infection [36]) in all eight pooled M-DBSs from Liberia. Furthermore, we identified signatures of CDV in the M-DBS pool from Village A d7 (Fig. 4) and we confirmed that this was the only pool containing CDV RNA by RT-PCR. Upon further inspection, only 1 of 57 M-DBSs from Village A d7 was positive for CDV and the sequenced 400 bp L gene (polymerase) amplicon was most similar to a South American isolate of CDV (97% nucleotide identity to GenBank accession number KM280689). Genetic material derived from porcine and canine origin was also identified in this pool by NGS. Using cytochrome b PCR, we identified that the M-DBS containing CDV RNA was the only bloodmeal from Village A d7 that was obtained from a dog. Isolation of either of the aforementioned viruses was not attempted because the FTA cards fully inactivate viruses [37,38]. Library quality and analysis accuracy were validated by the presence of reads aligning to *An*. *gambiae*, humans, and *Plasmodium falciparum* from all field-collected sample pools (S3 Fig.). The presence of *P*. *falciparum* in our samples would be expected because malaria is holoendemic in this region and the mosquito, the human upon which it fed, or both may be infected.

**Table 2:** Liberia mosquito collection summary^a^ and RNA-sequencing pools.

Pool ID	No. houses aspirated	No. mosquitoes collected	No. bloodfed mosquitoes collected	No. pooled M-DBS^c^	Total RNA recovered in 20 μl (μg)
Village A d1–2	21	271	193	31	5.20
Village A d3	11	342	268	38	4.00
Village A d4	7	117	99	24	6.56
Village A d5	12	427	361	41	10.78
Village A d6	10	290	251	29	4.62
Village A d7	15	719	582	57	6.64
Village B d1–2	11	249	200	51	7.00
Village B d3–4	11	312	225	57	9.88
Control^b^	NA	NA	NA	20	1.76

NA, not applicable. M-DBS, mosquito dried blood spots.

^a^All of the aspirated mosquitoes were identified by PCR as *An*. *gambiae* sensu stricto.

^b^Control pool generated from laboratory-raised *An*. *gambiae* mosquitoes that fed upon sheep’s blood.

^c^Up to four M-DBS from each house aspirated were pooled and used for RNA-sequencing.

**Table 3:** PathoScope reads alignment summaries.

			Reads aligned to:^b^					
Pool ID	Total reads	Reads after PathoQC	Mosquitoes	Humans	Other organisms	EBV^d^	CDV^e^	Reads not aligned^b^
Village A d1–2	53,027,225	36,983,929	22,001,739 (59.5%)	247,961 (0.7%)	69,661 (0.2%)	23	0	14,644,568 (39.7%)
Village A d3	48,375,950	32,605,957	19,573,355 (60%)	372,789 (1.1%)	63,827 (0.2%)	10	0	12,595,986 (38.6%)
Village A d4	49,354,744	32,955,955	20,218,478 (61.3%)	255,938 (0.8%)	72,562 (0.2%)	29	0	12,408,977 (37.7%)
Village A d5	48,863,196	34,338,484	20,348,985 (59.3%)	625,186 (1.8%)	54,452 (0.2%)	55	0	13,309,816 (38.8%)
Village A d6	55,890,046	37,353,434	21,541,725 (57.7%)	424,194 (1.1%)	272,949 (0.7%)	65	0	15,144,566 (40.5%)
Village A d7	45,952,027	33,509,057	18,084,838 (54%)	155,207 (0.5%)	1,188,449 (3.5%)	55	41	14,080,563 (42%)
Village B d1–2	56,584,512	39,927,794	22,271,723 (55.8%)	279,657 (0.7%)	66,592 (0.1%)	36	0	17,309,822 (43.4%)
Village B d3–4	41,312,457	31,739,159	17,053,450 (53.7%)	731,714 (2.3%)	619,781 (2%)	22	0	13,334,214 (42%)
Control^a^	36,825,109	29,667,973	16,433,090 (45.3%)	3616^c^ (0.01%)	1,043,076 (3.5%)	0	0	17,479,782 (58.9%)

EBV, Epstein-Barr virus; CDV, canine distemper virus.

^a^Control pool generated from laboratory-raised *An*. *gambiae* mosquitoes that fed upod sheep’s blood.

^b^Denotes percentage after PathoQC.

^c^Denotes reads aligning to the sheep reference library.

^d^Denotes reads aligned to EBV strain B95–8 (GenBank V01555.2)

^e^Denotes reads aligned to CDV strain Uy251 (GenBank KM280689.1)

## Discussion

Blood acquisition is an indispensable activity for the reproduction of most mosquito species. *An*. *gambiae* mosquitoes imbibe several times their body weight in blood, and they spend several hours immediately following bloodmeal acquisition undergoing the initial stages of bloodmeal digestion [39]. Consequently the mosquito midgut becomes a highly proteolytic environment that facilitates the degradation of the blood contents over approximately the first 24 hours after blood ingestion [40]. Previous studies have demonstrated that vertebrate pathogens, specifically viruses, can be detected in the bloodmeal of recently engorged mosquitoes [12–14], but the stability and recovery rates of viral RNA after prolonged exposure to the mosquito midgut was undefined. Therefore, we assessed our ability to recover WNV and HIV-1 RNA from *An*. *gambiae* and revealed that 12 hours exposure to the midgut environment did not significantly decrease the recovery of viral RNA. However, we did observe a significant reduction in WNV RNA recovery as a result of blood application onto FTA cards. Surprisingly, a similar reduction was not observed for HIV-1. This discrepancy might be explained by the relative stability of each of the viruses [41,42]. Alternatively, there may be differences in the efficiency of binding to and/ or elution from FTA cards between the two viral RNA species. These observations may need further examination to determine if biases are being introduced in future sampling efforts. While there are some discrepancies with viral RNA recovery from FTA cards, overall these findings demonstrate that viral RNA remains mostly protected from the highly proteolytic environment of the mosquito midgut and that mosquitoes can be used to sample viral pathogens in human beings.

*An*. *gambiae* are nocturnal, endophilic feeders that rest on interior walls for several hours after feeding. As a result engorged mosquitoes can be captured during the early morning hours via aspiration. The elapsed time between bloodmeal acquisition and collection can range anywhere from 4–36 hours post feeding, but is most likely within the 4–14 hpf time-frame. Because of this broad range of times, we assessed the efficacy of our approach over varying post-feeding durations. We found that we could reliably detect WNV RNA in extracted bloodmeals for up to 18 hpf and up to 24 hours for HIV-1. The aforementioned differences in viral stability may account for the observed discrepancy in our detection at 24 hpf [41, 42]. Together these findings suggest that viral RNA remains detectable in the mosquito midgut within a field-relevant time-frame.

Viral loads circulating in the blood are dynamic and constantly change during an active infection. In addition, viral loads can differ significantly between viral species. Therefore, we determined the detection limits for four different RNA viruses and compared our findings to viral loads in clinically apparent infections [8,30–34]. We observed significant differences in our ability to detect each of the four viruses with HIV-1 having the lowest 50% endpoint detection (~10^5^ RNA copies/ml) and CHIKV having the highest (~10^7^ RNA copies/ml). These large discrepancies between the viruses could in part be explained by potential differences in viral stability or binding to and/ or elution efficiency from FTA cards. Regardless, each of the 50% endpoint detections were well within the clinical ranges for each of the respective viruses [8,30–34]. While there may be greater chance to detect chronic viral infections (e.g. HIV-1), our data indicates that there is still a window to detect many acute stage viral infections with mosquitoes.

During viral infection, viruses are not only freely circulating in the blood, but also cell-associated (e.g. HIV in T-cells and macrophages). Because our artificial feeding approach does not accurately replicate this scenario, we assessed sensitivity using actively replicating virus in hamsters. This is important to determine because cell-associated viral RNA (immature virions or replicative intermediates) could impact the sensitivity of detection. Furthermore, it is unknown if and how cells may affect the stability of viral RNA in the context of the mosquito midgut. In our WNV- and PIRV-hamster models, we determined that bloodmeals derived from actively replicating infections do not significantly impact our ability to detect viral RNA using the xenosurveillance approach. These findings suggest that in the context of the xenosurveillance workflow, artificial membrane feeding of virus spiked blood accurately mimics an active infection thereby validating our *in vitro* laboratory findings.

Using PCR-based assays, we established the feasibility of xenosurveillance both in the laboratory and in the field in a pathogen-specific manner; however, there may be instances where an unbiased pathogen approach is required, such as the detection of under-represented, regionally unique, and/ or novel pathogens. NGS based metagenomics can be used to characterize the genomic landscape of complex biological or environmental samples without *a priori* knowledge of the microbial community [9]. Therefore, we determined if NGS-directed metagenomics approach could be used to detect pathogen-derived genetic signatures in field-collected samples. We found evidence of two potentially important animal viruses, EBV and CDV. EBV is a highly prevalent gamma-herpesvirus that infects B-lymphocytes and develops latency [36,43]. Because of its tissue tropism and its associated reactivation during *P*. *falciparum* infections, it is not surprising that latent EBV infections of white blood cells would be readily detectable by our approach in a *P*. *falciparum* holoendemic region such as Liberia [43]. CDV is a morbillivirus (related to measles virus) and an emerging virus of veterinary significance. Spillover of CDV from domestic dogs has caused mass mortality in endangered African carnivores [44–46]. Detection of CDV from one mosquito that fed on a dog in a pool of 57 M-DBSs is important for two reasons. First, it demonstrates the sensitivity of using NGS to screen for pathogen signatures in pooled M-DBS. Second, it suggests that xenosurveillance may be useful for the detection of veterinary or zoonotic pathogens in areas where indoor-resting mosquitoes prefer to feed on domestic animals [47,48]. Currently, the costs associated with NGS are impractically high for resource-poor regions; however, costs have been falling precipitously far outpacing the predictions associated with Moore’s Law [49]. In addition to NGS, we demonstrated that viruses and bloodmeal sources could also be detected by more cost efficient PCR-based methods from pooled and individual M-DBSs, further broadening the utility of xenosurveillance.

Biosurveillance is the process of collecting, analyzing, and interpreting biosphere data in order to provide early detection of biological threats to human or animal health. However this process can be labor intensive, complex, costly, and ineffective, particularly in under-developed and remote locales such as tropical Africa. In this manuscript we describe the use of a novel sample acquisition and processing strategy termed xenosurveillance that can be used to improve the collection, storage, and shipment of biological samples from the field. Collecting bloodfed mosquitoes from houses is relatively easy, inexpensive and noninvasive compared to finger-pricks or venous blood draws. These factors may make it ideal for longitudinal sampling surveys and identification of viruses that induce transient viremias. In our experience, villagers welcome taking mosquitoes from their houses frequently, which may reduce their risk of future infections from vector-borne pathogens like *Plasmodium*. Consent from the head-of-households for mosquito collections is required, but xenosurveillance is not equivalent to sampling blood from patients directly, which requires individual informed consent, because the exact source of any bloodmeal cannot be identified without a matching blood sample from a patient. This is because up to 10% of *An*. *gambiae* bloodmeals are acquired from individuals living outside the home in which the mosquito was captured and up 20% have acquired bloodmeals from two or more people [11]. Nonetheless, xenosurveillance identification of the genetic signatures of certain pathogens (eg. HIV) should require, at minimum, notification to the local public health authorities of the possibility of the pathogen circulating in the area of the sampling. Aside from sample acquisition, xenosurveillance also offers benefits when processing samples. For instance, this approach offers increased portability because very few tools/ reagents are required and FTA cards eliminate the need for a cold-chain [15–17]. Moreover, the chemical properties associated with FTA cards completely inactivates viruses as has been previously demonstrated for two divergent enveloped RNA viruses, porcine reproductive and respiratory syndrome virus (*Arteriviridae*) and Newcastle disease virus (*Paramyxoviridae*) [37,38], thereby increasing the safety of handling and simplifying international shipping. While we specifically addressed the utility of this workflow in the context of viral pathogens, the procedure could be applied to any pathogen found circulating in the blood of humans and other animals (although the sensitivity and duration of detection still need to be validated for non-virus pathogens). We have demonstrated the feasibility of this approach and determined the limits of detection using qRT-PCR on individual M-DBS as well as identified vertebrate viral pathogens from field-caught mosquitoes. Regardless of the bio-detection technology utilized, such samples can be used for the early detection of recent spillover events or of pathogens with epidemic potential. Furthermore, previous studies suggest that this approach could be used to complement traditional sero-epidemiological studies [50,51]. Finally, while this study exclusively examined the utility of mosquitoes for the purpose of xenosurveillance, there is no reason to believe that other hematophagous insects or other animals (e.g. leeches) cannot be used for similar purposes [52,53]. Together, this approach provides an attractive alternative to conventional approaches for epidemiological and surveillance studies of human pathogens in remote tropical locations.

## Supporting Information

S1 Fig Schematic representation for collection and storage of bloodmeals from of indoor resting, naturally bloodfed mosquitoes. (A) Indoor resting mosquitoes are aspirated, (B) transferred into cartons by house, (C) anesthetized by triethylamine, (D) sorted by blood feeding status and morphologically identified, and (E) the bloodmeals were applied to FTA cards. (F) The FTA cards were stored in multi-barrier pouches containing desiccant in a cool, dry place for up to 3 weeks until transportation.
(TIF)

S2 Fig Comparisons of using PBS or ethanol from bloodmeal application onto FTA cards. Mosquito bloodmeals containing 10^6^ GE/ml of HIV-1 RNA were applied to FTA cards using either 5μl of PBS or ethanol (EtOH) to help diffuse to blood. The methods were compared by the recovery of (A) HIV-1 RNA and (B) total RNA extracted from the M-DBSs (unpaired T-tests).
(TIF)

S3 Fig The microbial composition of NGS reads from field-collected mosquito bloodmeals determined by PathoScope. Mosquito bloodmeals were collected from village A and village B every 3 days and every 5 or 6 days, respectively, over a course of three weeks in northern Liberia. NGS was performed on RNA pooled from 20–51 M-DBS per collection and the sequencing reads were aligned to the microbial reference libraries using PathoScope.
(TIF)

S1 Table Primers and probes for qRT-PCR quantification of RNA copies. (DOCX)

S2 Table Summaries of the genome reference libraries. (DOCX)

S3 Table PathoScope parameter values used in this study. (DOCX)
