# Supplementary figures and images for: Xenosurveillance: A Novel Mosquito-Based Approach for Examining the Human-Pathogen Landscape
Source: PLoS Negl Trop Dis. 2015 Mar 16;9(3):e0003628. doi: 10.1371/journal.pntd.0003628 (PMC4361501; doi:10.1371/journal.pntd.0003628)

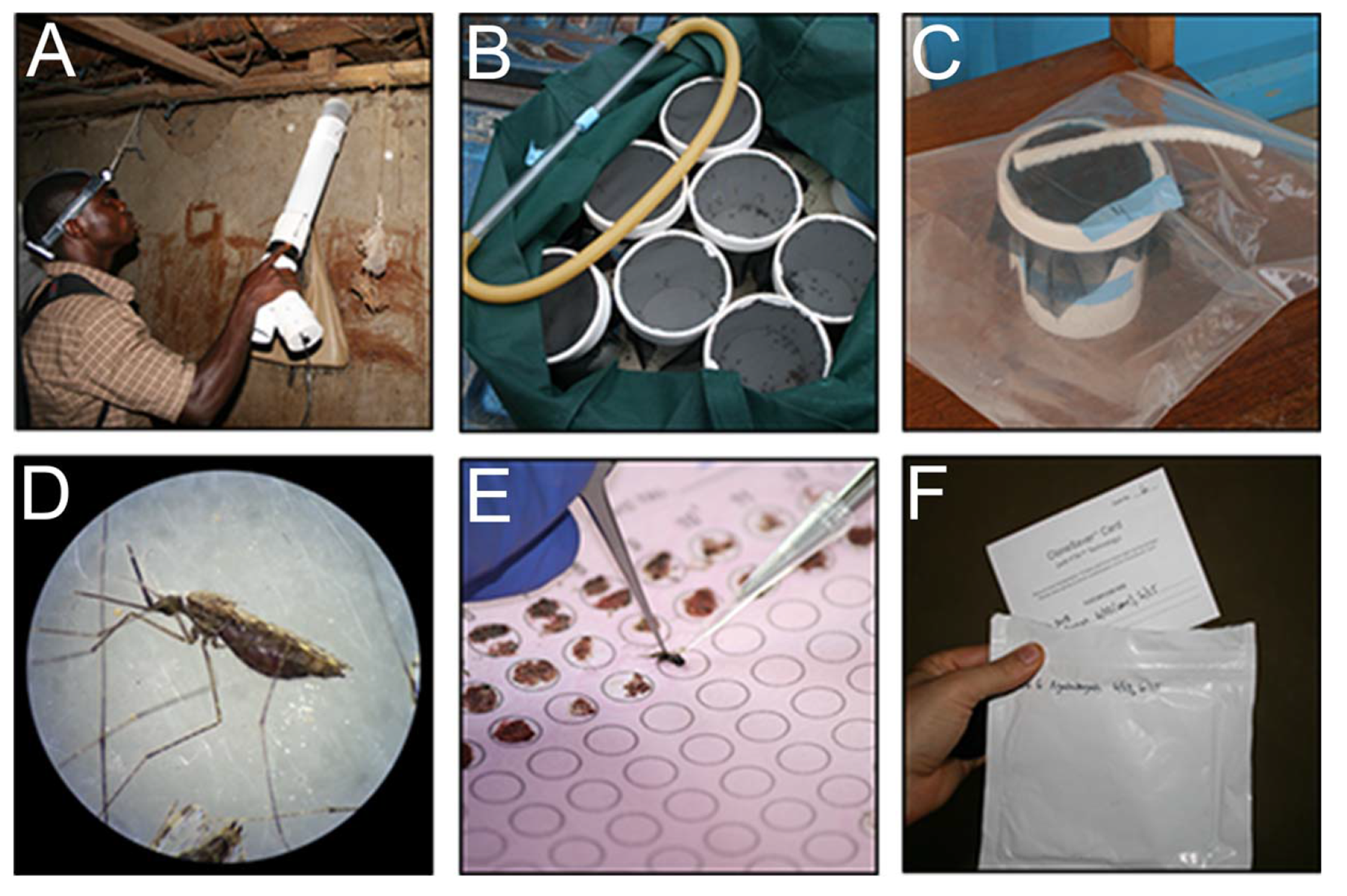

Supplement: S1 Fig — (A) Indoor resting mosquitoes are aspirated, (B) transferred into cartons by house, (C) anesthetized by triethylamine, (D) sorted by blood feeding status and morphologically identified, and (E) the bloodmeals were applied to FTA cards. (F) The FTA cards were stored in multi-barrier pouches containing desiccant in a cool, dry place for up to 3 weeks until transportation. (TIF) [file pntd.0003628.s001.tif]

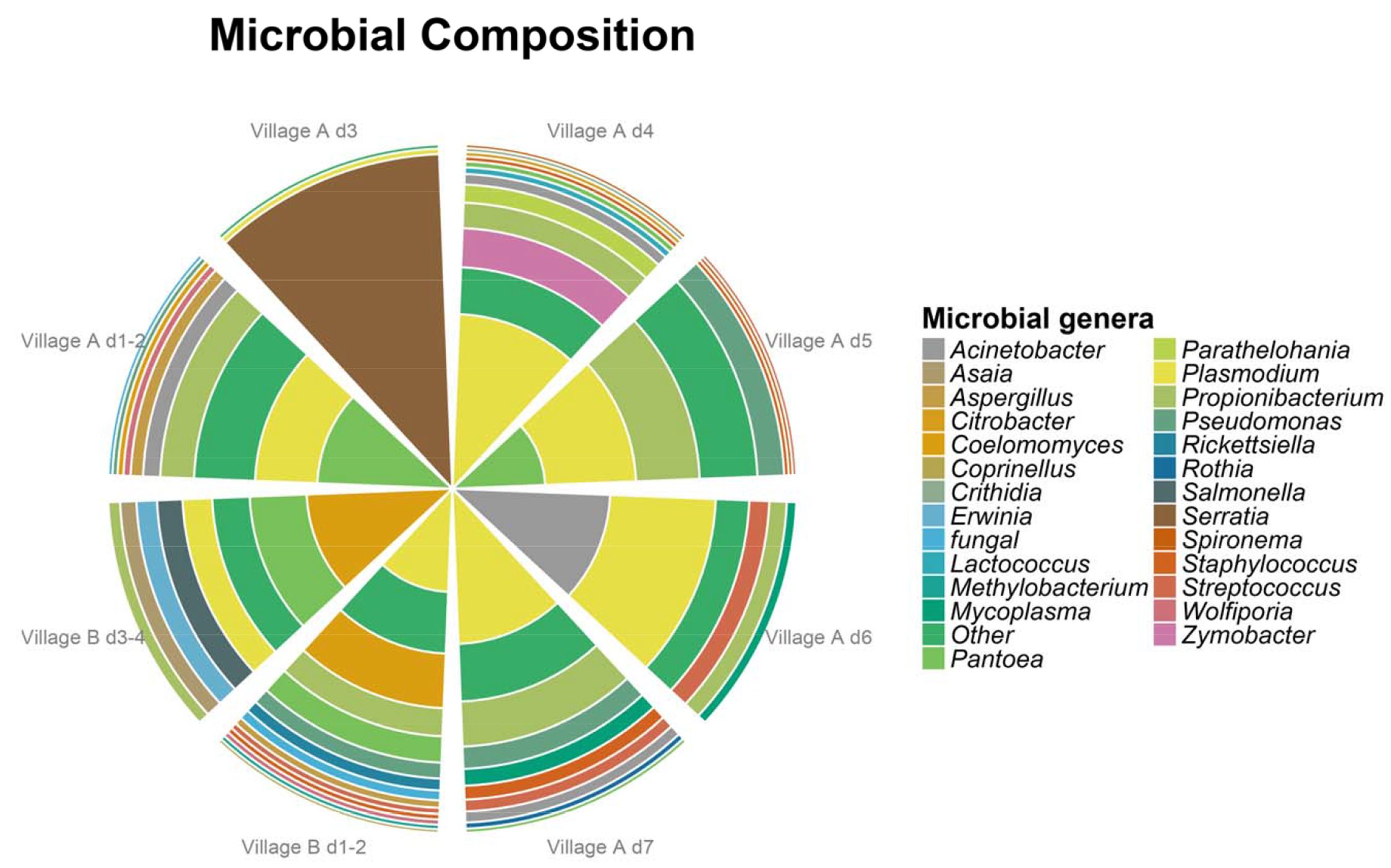

Supplement: S3 Fig — Mosquito bloodmeals were collected from village A and village B every 3 days and every 5 or 6 days, respectively, over a course of three weeks in northern Liberia. NGS was performed on RNA pooled from 20–51 M-DBS per collection and the sequencing reads were aligned to the microbial reference libraries using PathoScope. (TIF) [file pntd.0003628.s003.tif]
