# Supplementary material for: Xenosurveillance: A Novel Mosquito-Based Approach for Examining the Human-Pathogen Landscape
Source: PLoS Negl Trop Dis. 2015 Mar 16;9(3):e0003628. doi: 10.1371/journal.pntd.0003628 (PMC4361501; doi:10.1371/journal.pntd.0003628)
Supplement: S1 Table — (DOCX) [file pntd.0003628.s004.docx]

|  | **Table S1.** Primers and probes for qRT-PCR quantification of RNA copies | | | | | |
| --- | --- | --- | --- | --- | --- | --- |
| **Virus/gene** | | **Primer/**  **probe** | **Sequence (5’ to 3’)** | **Position (nt)** | **qRT-PCR efficency** |  |
| HIV-1 gag | | sense | AGA CAA GGA CCA AAG GAA CC | 1191-1210 | 82.8% |  |
|  | | antisense | AGT CTT ACA ATC TGG GTT CGC | 1331-1311 |  |  |
|  | | probe^a^ | AAC TCT AAG AGC CGA GCA AGC TTC AC | 1241-1266 |  |  |
| WNV E^n^ | | sense | TCA GCG ATC TCT CCA CCA AAG | 1160-1180 | 99.1% |  |
|  | | antisense | GGG TCA GCA CGT TTG TCA TTG | 1229-1209 |  |  |
|  | | probe^a^ | TGC CCG ACC ATG GGA GAA GCT C | 1186-1207 |  |  |
| PIRV nc | | sense | CTG TGC AGT GAG AAC AAT TCC TG | 1959-1981 | 93.5% |  |
|  | | antisense | TGC TAC AGG GAG CCC CAT GA | 2111-2089 |  |  |
|  | | probe^a^ | CCC AGG TTG TGT GTT TGT AAG | 2013-2032 |  |  |
| CHIKV ns2B | | sense | CAC CTG AAG TAG CCC TGA ATG | 3220-3240 | 88.3% |  |
|  | | antisense | TCC GAA CAT TTT CCC TCC AG | 3359-3340 |  |  |
|  | | probe^a^ | AAA ATA GCC CGC TGT CTA GAT CCA CC | 3288-3313 |  |  |
| EBV EBER2 | | sense | TTT GCA AGT CAG GAT TCT CT | 7067-7086 | NA |  |
|  | | antisense | TAA CTG GGT GTC TAC CTG AA | 7257-7257 |  |  |
| CDV L | | sense | TGA CAA CAT ACA AGC TAG GC | 13842-13861 | NA |  |
|  | | antisense | GTG TGT CCT TGT TGA TAG CT | 14222-14241 |  |  |

HIV-1 gag, human immunodeficiency virus 1 group-specific antigen gene; WNV E, West Nile virus envelope gene; PIRV nc, Pirital virus S segment nucleocapsid gene; CHIKV ns2B, chikungunya virus nonstructural 2B gene; EBV EBER-2, Epstein-Barr virus-encoded RNA 2; CDV L, canine distemper virus large gene.

^a^5’ end of probe labeled with a FAM (6-carboxyflourescein) flourophore reporter molecule and ZEN-Iowa Black FQ double quenchers (Integrated DNA Technologies Inc., San Jose, CA)

^b^see reference [23]
