# Supplementary material for: Xenosurveillance: A Novel Mosquito-Based Approach for Examining the Human-Pathogen Landscape
Source: PLoS Negl Trop Dis. 2015 Mar 16;9(3):e0003628. doi: 10.1371/journal.pntd.0003628 (PMC4361501; doi:10.1371/journal.pntd.0003628)
Supplement: S2 Table — (DOCX) [file pntd.0003628.s005.docx]

| Table S2. Summaries of the genome reference libraries. | | | | |
| --- | --- | --- | --- | --- |
| Reference library name | **Parent taxonomy ID** | **Genomes represented** | **Number of sequence fragments** | **Total number of bases** |
| Mosquito | 7157 | 792 | 163,171 | 165,408,816 |
| Human | 9606 | 3 | 670,844 | 5,710,985,516 |
| Pig | 9823 | 12 | 92,192 | 408,480,321 |
| Dog | 9608 | 65 | 36,254 | 141,595,637 |
| Goat | 9925 | 1 | 8,412 | 6,389,904 |
| Sheep | 9940 | 3 | 33,423 | 62,921,654 |
| Bacteria | 2 | 262,010 | 4,408,546 | 12,054,623,913 |
| Fungus | 4751 | 83,676 | 1,727,050 | 2,401,824,484 |
| Kinetoplastida | 5653 | 938 | 94,220 | 389,825,747 |
| Nematoda | 6231 | 4,950 | 195,453 | 310,259,835 |
| Protostomia | 33317 | 142,200 | 1,906,119 | 2,403,562,421 |
| Plasmodium | 5820 | 1,145 | 96,199 | 171,603,707 |
| Virus | 10239 | 97,251 | 1,337,444 | 1,750,565,215 |
