# Supplementary material for: Xenosurveillance: A Novel Mosquito-Based Approach for Examining the Human-Pathogen Landscape
Source: PLoS Negl Trop Dis. 2015 Mar 16;9(3):e0003628. doi: 10.1371/journal.pntd.0003628 (PMC4361501; doi:10.1371/journal.pntd.0003628)
Supplement: S3 Table — (DOCX) [file pntd.0003628.s006.docx]

| **Table S3.** PathoScope parameter values used in this study. | |
| --- | --- |
| **Method** | **Parameter Settings** |
| PathoLib | Mosquito - taxonID 7157 Human - taxonID 9606  Pig – taxoID 9823 Dog – taxoID 9608  Goat – taxoID 9925 Sheep - taxoID 9940  Bacteria - taxonID 2 Fungus - taxoID 4751  Kinetoplastida - taxonID 5653 Nematoda - taxoID 6231  Protostomia - taxoID 33317 Plasmodium - taxoID 5820  Virus - taxoID 10239 |
| PathoQC | -t 33, -m 25, -e 50, -g 1, -d 1, -q 0, -p 4, -a Y |
| PathoMap | -TargetAlignParams "--very-sensitive-local -k 500 --score-min L,205,0.0" |
| PathoID | Default settings |
| PathoReport | --contig |
